# Food for thought: re-emergence of tardive dyskinesia amid nutritional rehabilitation of atypical anorexia nervosa; a case report

**DOI:** 10.1186/s40337-025-01466-w

**Published:** 2025-11-26

**Authors:** Nicole Gulino, Ally Reardon, Matthew J. Johnson

**Affiliations:** 1 Rutgers Robert Wood Johnson Medical School, 125 Paterson St, New Brunswick, NJ 08901 USA; 2 Department of Psychiatry, Robert Wood Johnson University Hospital-Somerset, 110 Rehill Ave, Somerville, 08876 NJ USA; 3 Encompass Psychiatry, Morristown, NJ USA

**Keywords:** Case report, Tardive dyskinesia, Lurasidone, Anorexia, Nutritional rehabilitation

## Abstract

**Background:**

Atypical anorexia nervosa is an eating disorder that involves significant restriction of food intake leading to clinically significant weight loss despite remaining at a normal or above normal weight for age or height. Part of rehabilitation involves increasing calorie intake for nutritional rehabilitation and weight gain to an appropriate percentage of ideal body weight, determined based on patient weight history. This report highlights the importance of considering how increased food intake in a previously malnourished patient can affect the metabolism of medications they are taking for the management of co-occurring psychiatric conditions, including the possibility of the emergence or re-emergence of tardive dyskinesia for a patient on an antipsychotic.

**Case presentation:**

This case presents a 43-year-old female with a history of bipolar disorder type 1, controlled on lurasidone, admitted to an inpatient eating disorder unit for nutritional rehabilitation and weight restoration for atypical anorexia nervosa. Shortly after admission, despite no initial change in medication dosing, the patient began exhibiting symptoms of tardive dyskinesia. These symptoms were reduced once her lurasidone dose was decreased.

**Conclusions:**

Nutritional rehabilitation likely has an impact on the metabolism of certain antipsychotic medications including lurasidone. More research is needed involving patients recovering from a restrictive eating disorder who are on antipsychotic medications and the frequency of increased side effects, including tardive dyskinesia, with refeeding.

## Background

Atypical anorexia nervosa (AAN) is an eating disorder characterized by significant energy restriction leading to clinically significant weight loss despite remaining at a normal or above normal weight for age or height [1]. Anorexia nervosa (AN) presents similarly, but with a reduction in body weight leading to low body weight and body mass index (BMI) for age or height [2]. In both conditions, current management strategies focus on nutritional rehabilitation and weight gain to an appropriate percentage of ideal body weight, determined by the patient’s weight history. Pharmacologic agents, such as antidepressants, antipsychotics, anti-anxiety medications, and psychedelics may at times be used as adjunctive treatment [3]. Regarding the use of antipsychotics, research has focused on olanzapine for AN, with multiple reviews excluding lurasidone as a pharmacological intervention [3–5]. In our review of the literature, no studies were found that looked at pharmacological intervention on AAN specifically.

Tardive dyskinesia (TD) is an involuntary movement disorder caused by the blocking of dopamine receptors and is commonly implicated by starting, discontinuing, or changing the dosage of psychotropic medication [2]. To our current knowledge, there is no data connecting TD and AN outside of a 1986 case report where a patient began to exhibit signs following high dose neuroleptic therapy [5, 6]. Similarly, no data was found connecting TD or AAN. Current research suggests that drug metabolism is altered by changes in BMI, which can be seen during nutritional rehabilitation, specifically in regards to CYP3A4 [7–9]. Limited research has also suggested that refeeding and nutritional rehabilitation have the potential to impact pharmacokinetics and drug metabolism [10, 11]. However, there is no current research reporting a connection between AN or AAN and TD, especially in the setting of nutritional rehabilitation. We report a case of a 43-year-old female with AAN that experienced reemergent TD despite stable dosage of lurasidone, potentially due to the changes in drug metabolism following nutritional rehabilitation. The patient gave permission to publish the following case report.

## Case presentation

The patient is a 43-year-old female with a past medical history of bipolar disorder type 1, post-traumatic stress disorder (PTSD), attention-deficit hyperactivity disorder (ADHD), and AAN who presented for evaluation for her eating disorder. She had previously been psychiatrically hospitalized as a child following a sexual assault and later as an adult for suicidal ideations. She has also attended treatment for her eating disorder at both residential and inpatient facilities in the past. Family psychiatric history includes bipolar disorder and ADHD. She was diagnosed with bipolar disorder type 1 with prominent manic episodes with psychosis and was started on antipsychotic medication shortly thereafter. Approximately 9 years after starting treatment, she then started to experience TD symptoms, 2 years prior to presentation. Symptoms were noted by her psychiatrist at the time as clenching and moving the jaw unintentionally. She was started on valbenazine 40 mg shortly thereafter. This improved her symptoms but did not resolve them completely so the dose was adjusted to 80 mg a couple months later with symptoms only returning intermittently and mildly during times of high stress. Lurasidone was added to her medication regimen at 60 mg per day a few months later for further mood stabilization, approximately 6 months before presentation.

Her medication regimen leading up to admission included:

- lisdexamfetamine 70 mg daily AM
- armodafinil 200 mg daily AM
- memantine 5 mg BID
- lumateperone 42 mg nightly
- lurasidone 60 mg with lunch
- amitriptyline 50 mg nightly
- valbenazine 80 mg daily AM
- lorazepam 1 mg TID PRN anxiety

All medications were started prior to admission; no new medications or dose adjustments were made to psychiatric medications at the time of admission as her bipolar disorder and tardive dyskinesia were well-controlled and considered to be in remission.

On her psychiatric evaluation on hospital day (HD) 1, the patient was not exhibiting any signs of TD including no tics or abnormal movements. Over the next month, her meal plan was gradually increased to an appropriate target to allow for weight restoration and nutritional rehabilitation. On HD 32, symptoms of TD re-emergence were initially noted which included involuntary tongue movements, teeth grinding, and abdominal crunching in the supine position. Her current medication regimen at this time is listed below, with new medications and supplements added since admission in bold:

- armodafinil 200 mg Daily
- **Bariatric Multicapsule Daily**
- **docusate sodium 200 mg**,** oral**,** BID**
- **folic acid 1 mg Daily**
- lisdexamfetamine 70 mg Daily
- lumateperone 42 mg Daily
- lurasidone 60 mg, oral, Daily
- **megestroL 400 mg/10 mL (10 mL) suspension 800 mg**,** oral**,** Daily**
- memantine 5 mg, oral, BID
- **pregabalin 50 mg TID**
- **rifAXIMin 550 mg TID**
- **sodium chloride soluble tablet 1 g BID with meals**
- **thiamine 100 mg Daily**
- valbenazine 80 mg Nightly

As symptoms persisted on HD 33, her dose of lurasidone was adjusted from 60 mg to 40 mg for HD 34 onward. A decrease in TD symptoms was noted already on HD35, but they had not resolved. As symptoms continued to slowly dissipate over the following days, the patient was then discharged from the EDU on HD 41 for continued outpatient management. See table below for the summary:

**Table 1:** TD symptoms and nutritional rehabilitation timeline

Date	Event
HD 0	EDU admission. Leading up to admission, patient reported eating 200–500 kcals per day for several months
HD 1	No TD movements or tics noted on the physical exam. Lurasidone 60 mg daily was continued for mood stabilization. Meal plan was started at 1500 kcal per day
HD 14	Meal plan increased to 1800 kcal per day
HD 28	Meal plan increased to 2100 kcal per day
HD 32	Initial re-emergence of TD symptoms
HD 33	Meal plan increased to 2400 kcal per day
HD 34	New dose of lurasidone (40 mg daily) was started
HD 35	Decrease in TD symptoms were noted, but they were not resolved
HD 40	Meal plan increased to 2700 kcal per day
HD 41	EDU discharge

On follow-up with the patient, she was switched from valbenazine to deutetrabenazine soon after discharge from the EDU and gradually titrated from the lowest dose to the maximal dose. This titration occurred over nearly 12 months, and per patient symptoms finally improved significantly by July 2025 and now only occur mildly with increased stress.

## Discussion and conclusions

This case illustrates how nutritional rehabilitation and increased caloric intake in a malnourished patient on an antipsychotic may affect the pharmacokinetics and pharmacodynamics of this type of medication. TD most commonly occurs in patients on long-term medications that are dopamine antagonists [12]. TD can also present when these medications are either increased or decreased, especially rapidly. Lurasidone (a second- generation antipsychotic) was the main medication the patient was on that decreased dopamine levels. It was most likely that the original appearance of TD symptoms for this patient was triggered by long-term use of an anti-psychotic similar to lurasidone for 9 years. She was also on lumateperone, which mainly blocks serotonin receptors but is a partial agonist at presynaptic dopamine receptors and a partial antagonist at postsynaptic dopamine receptors [13]. While rates of TD are reported to be lower with lumateperone compared to those for the second-generation antipsychotics such as lurasidone, it’s still noted to be a potential side effect. Neither of the doses for these medications were changed leading to her admission or during admission before TD symptoms re-emerged, so these medications are unlikely to have caused symptoms to re-emerge on their own. Notably, research has found that anti-emetics have a strong potential to induce tardive dyskinesia, especially metoclopramide and prochlorperazine, due to their antagonistic effects on the dopamine (D2) receptor, and these medications are commonly used in the eating disorder recovery setting [12]. However, upon chart review our patient was not taking any scheduled or PRN anti-nausea medications during the time of symptom re-emergence. It is worth noting that the patient was started on megestrol for appetite stimulation at a dose of 800 mg oral suspension on HD 21. This medication is a known inducer of the CYP3A4 enzyme [14], the same enzyme that metabolizes lurasidone. However, an inducer would increase the rate of metabolism of drugs using the CYP3A4 enzyme, which would decrease the peak serum concentrations of lurasidone. While it is possible that a decrease in an antipsychotic medication can cause TD side effects, the fact that decreasing the patient’s dose of lurasidone later improved symptoms makes it less likely that the addition of megestrol contributed to the symptom re-emergence, and in fact it makes it more likely that the nutritional rehabilitation could have played a potentially larger influence without the addition of megestrol.

There is evidence that the presence of food in the stomach or small bowel can increase the rate of absorption of certain medications, including lurasidone, and that as the calorie content of the meal increases, so does the Cmax (or peak plasma concentration) [15]. Per the FDA label [16], Lurasidone should be taken with food, at least 350 calories, as it substantially increases the absorption, doubling the AUC, and tripling the Cmax. Of note, the absorption of most psychiatric medications is either unaffected by or decreased by the presence of food in the stomach, but there are a few common psychiatric medications that require a bolus of food for optimal absorption [17], which are illustrated below:

**Table 2:** Psychiatric medications requiring a food bolus for optimal absorption

Medication	Recommended intake/effect of food
Lurasidone	*≥* 350 kcal
Ziprasidone	*≥* 500 kcal
Paliperidone	Absorption drops by 50–60% on an empty stomach
Vilazadone	Absorption drops 50% on an empty stomach
Gabapentin XR	Absorption drops 50% on an empty stomach

On review of this patient’s chart, the lurasidone dose was typically given within 30–60 min of completing a meal. As the calorie content of her meals increased during her admission, it’s possible that this gradually allowed the serum concentration of lurasidone to build up to a level that resulted in the re-emergence of TD symptoms. This theory is supported by the reported decrease in TD symptoms after reducing her lurasidone dose from 60 mg to 40 mg. However, there is limited literature available on the effects of refeeding for individuals with AAN or other restrictive eating disorders and the emergence or re-emergence of TD when patients are on an antipsychotic medication.

This case suggests that for clinicians managing nutritional rehabilitation of patients with a restrictive eating disorder, monitoring for side effects of antipsychotic medications when prescribed or continued on admission is warranted. It is likely that for certain medications, including lurasidone, dosing will need to be adjusted as food intake increases. More research is needed to make specific recommendations on how and when dose adjustments should be considered for individuals in recovery from a restrictive eating disorder.

## Data Availability

No datasets were generated or analysed during the current study.

## Abbreviations

AAN: Atypical anorexia nervosa
ADHD: Attention-deficit hyperactivity disorder
AN: Anorexia nervosa
BMI: Body mass index
EDU: Eating disorder unit
PTSD: Post-traumatic stress disorder
TD: Tardive dyskinesia
HD: Hospital day
